# Exploring the transcriptomic landscape of moyamoya disease and systemic lupus erythematosus: insights into crosstalk genes and immune relationships

**DOI:** 10.3389/fimmu.2024.1456392

**Published:** 2024-09-03

**Authors:** Qingbao Guo, Yan-Na Fan, Manli Xie, Qian-Nan Wang, Jingjie Li, Simeng Liu, Xiaopeng Wang, Dan Yu, Zhengxing Zou, Gan Gao, Qian Zhang, Fangbin Hao, Jie Feng, Rimiao Yang, Minjie Wang, Heguan Fu, Xiangyang Bao, Lian Duan

**Affiliations:** ^1^ Medical School of Chinese PLA, Beijing, China; ^2^ Department of Neurosurgery, The First Medical Centre, Chinese PLA General Hospital, Beijing, China; ^3^ Department of Neurosurgery, The Fifth Medical Centre, Chinese PLA General Hospital, Beijing, China; ^4^ Department of Radiation Oncology, Senior Department of Oncology, The Fifth Medical Center of PLA General Hospital, Beijing, China; ^5^ Department of Occupational Diseases, Xi’an Central Hospital, Xi’an, Shanxi, China; ^6^ Department of Neurosurgery, The Eighth Medical Centre, Chinese PLA General Hospital, Beijing, China

**Keywords:** systemic lupus erythematosus, moyamoya disease, transcriptomic analysis, crosstalk genes, immune infiltration

## Abstract

**Background:**

Systemic Lupus Erythematosus (SLE) is acknowledged for its significant influence on systemic health. This study sought to explore potential crosstalk genes, pathways, and immune cells in the relationship between SLE and moyamoya disease (MMD).

**Methods:**

We obtained data on SLE and MMD from the Gene Expression Omnibus (GEO) database. Differential expression analysis and weighted gene co-expression network analysis (WGCNA) were conducted to identify common genes. Subsequently, Gene Ontology (GO) and Kyoto Encyclopedia of Genes and Genomes (KEGG) pathway enrichment analyses were performed on these shared genes. Hub genes were further selected through the least absolute shrinkage and selection operator (LASSO) regression, and a receiver operating characteristic (ROC) curve was generated based on the results of this selection. Finally, single-sample Gene Set Enrichment Analysis (ssGSEA) was utilized to assess the infiltration levels of 28 immune cells in the expression profile and their association with the identified hub genes.

**Results:**

By intersecting the important module genes from WGCNA with the DEGs, the study highlighted *CAMP, CFD, MYO1F, CTSS, DEFA3, NLRP12, MAN2B1, NMI, QPCT, KCNJ2, JAML, MPZL3, NDC80, FRAT2, THEMIS2, CCL4, FCER1A, EVI2B, CD74, HLA-DRB5, TOR4A, GAPT, CXCR1, LAG3, CD68, NCKAP1L, TMEM33*, and *S100P* as key crosstalk genes linking SLE and MMD. GO analysis indicated that these shared genes were predominantly enriched in immune system process and immune response. LASSO analysis identified *MPZL3* as the optimal shared diagnostic biomarkers for both SLE and MMD. Additionally, the analysis of immune cell infiltration revealed the significant involvement of activation of T and monocytes cells in the pathogenesis of SLE and MMD.

**Conclusion:**

This study is pioneering in its use of bioinformatics tools to explore the close genetic relationship between MMD and SLE. The genes *CAMP, CFD, MYO1F, CTSS, DEFA3, NLRP12, MAN2B1, NMI, QPCT, KCNJ2, JAML, MPZL3, NDC80, FRAT2, THEMIS2, CCL4, FCER1A, EVI2B, CD74, HLA-DRB5, TOR4A, GAPT, CXCR1, LAG3, CD68, NCKAP1L, TMEM33*, and *S100P* have been identified as key crosstalk genes that connect MMD and SLE. Activation of T and monocytes cells-mediated immune responses are proposed to play a significant role in the association between MMD and SLE.

## Introduction

Moyamoya disease is a progressive cerebrovascular disorder characterized by chronic blockage of the major arteries in the brain, leading to the development of a network of tiny blood vessels that attempt to compensate for the reduced blood flow. It is a rare condition affecting about 1 in 1,000,000 individuals, marked by ischemic events and a myriad of neurological symptoms, such as strokes and transient ischemic attacks (1). While the precise etiology of moyamoya disease remains unknown, a combination of genetic and environmental factors is believed to contribute to its onset and progression (2). While the exact pathogenesis of MMD is still under investigation, emerging evidence suggests that immune mechanisms play a pivotal role in its development and progression (3). This includes the involvement of inflammatory cytokines, autoimmune reactions, as well as genetic predispositions that may influence immunopathological responses (4). Abnormal immunological activity, such as the presence of circulating autoantibodies and elevated levels of pro-inflammatory cytokines, has been documented and correlated with disease activity in Moyamoya patients (5).

Systemic Lupus Erythematosus (SLE) is a highly prevalent autoimmune disease with a significant impact on patient morbidity and mortality (6). The etiology of SLE is multifaceted, with genetic, environmental, hormonal, and immunological factors contributing to its complex pathogenesis (7). Notably, infections have been posited to play a crucial role in the development and exacerbation of SLE (8). Epidemiologically, it is observed that infections can precipitate the onset of SLE and exacerbate its symptoms, particularly those infections that stimulate a robust immune response. SLE is considered to be associated with aberrations in immune regulation that lead to autoantibody production and the formation of immune complexes (9), which can be further stimulated by interactions with pathogens. Various viral and bacterial antigens have been implicated in the disease process, though the precise mechanisms remain to be fully elucidated (10, 11). Among the infectious agents, the Epstein-Barr virus (EBV) has received attention due to its ubiquitous nature and its ability to establish life-long latent infections, which may contribute to the chronic stimulation of the immune system in SLE patients (12). Furthermore, the hygiene hypothesis suggests a link between increased hygiene and autoimmune disease prevalence, potentially implicating the role of commensal microbes and the immune system’s development and regulation (13). The relationship between infections, immunity, and SLE supports the notion that managing chronic infections could be beneficial for patients with SLE. Indeed, targeted immunosuppressive therapies such as glucocorticoids and immunomodulators are central to SLE treatment regimens (14). These treatments aim to dampen the overactive immune response and reduce the formation of damaging immune complexes associated with SLE pathology.

Many case reports have examined the association of MMD with SLE (15–17), and MMD is more common in women (18), which is consistent with SLE (19). However, the exact crosstalk genes have not been elucidated yet. Therefore, there is a need for additional research to better understand the connection between MMD and SLE, particularly at the cellular and molecular levels. Given the advancements in microarray and high-throughput sequencing technologies, bioinformatics tools are increasingly being utilized to investigate the interplay between various diseases. In this study, we utilized bioinformatics techniques to identify potential shared genes between MMD and SLE and to assess how these genes interact with immune cells that infiltrate the affected tissues. This approach aims to deepen our understanding of the underlying pathophysiological mechanisms that could link MMD and SLE.

## Materials and methods

### Data download and processing

Gene expression profiles specific to MMD and SLE were procured from the Gene Expression Omnibus (GEO) repository. Detailed information about the datasets is available online at the GEO website: https://www.ncbi.nlm.nih.gov/geo/. The datasets were obtained in the MINiML format. Utilizing the GPL16699 platform, the GSE157628 and GSE141025 dataset consists of 36 subjects, with 19 classified as “diseased” and 17 as “control”. To assess diagnostic efficiency, we downloaded the GSE189993 dataset based on GPL16699 consists of 36 subjects, with 21 classified as “diseased” and 11 as “control”.

Gene expression datasets exploring SLE (GSE78193) employed the GPL6480 and GPL6848 platform and encompassed 125 samples, including 101 from SLE patients and 24 from healthy individuals serving as controls. In addition to this dataset, GSE154851 was acquired—utilizing the GPL16699 platform—it comprised 38 SLE patient samples alongside 32 control samples, facilitating the evaluation of diagnostic accuracy. The detailed information regarding the datasets included in this study is available in **Table 1**.

**Table 1:** Detailed information about the datasets used in the study.

Dataset Name	Sample Type	Cases	Controls	Sex(F)	Age(y)
GSE157628	Micro-samples of the MCA from MMD and IA	11	9	16 (80.0%)	54 ± 17
GSE141025	Intracranial Artery from MMD and IA	8	8	14 (87.5%)	61 ± 13
GSE189993	Micro-samples of MCA from MMD, IA, and EPI	21	11	22 (68.8%)	44 ± 20
GSE78193	Blood samples from SLE and healthy volunteers	101	24	none	none
GSE154851	Blood samples from SLE and healthy volunteers	38	32	68 (97.1%)	35 ± 11

MMD, moyamoya disease; IA, internal carotid artery aneurysm; EPI, epilepsy; SLE, systemic lupus erythematosus.

Data normalization is a crucial step to ensure the comparability and reliability of data by removing biases and inconsistencies between samples. In this study, we employed various normalization techniques to standardize the data. We applied Z-score normalization, where each feature’s values were transformed by subtracting the mean and dividing by the standard deviation. This transformation resulted in a standard normal distribution of the data, reducing the impact of outliers and ensuring uniform scaling across features. To address potential batch effects in our dataset, we implemented Uniform Manifold Approximation and Projection (UMAP) as a batch correction method. Data normalization and batch effect processing in this study are detailed in **Supplementary Figure S1**.

### Detection of differentially expressed genes

Differential expression analysis is a critical aspect of our study, aimed at identifying genes that are differentially expressed between two or more groups of samples. To perform this analysis, we used the Linear Models for Microarray Data (LIMMA) package (version 3.32.4) within the R software. The approach taken using LIMMA is designed to model the expression data efficiently, taking into account the various sources of biological and technical variability. The “LIMMA” R package was employed to identify DEGs in the GSE157628, GSE141025, and GSE78193 datasets. DEGs were determined in GSE157628, GSE141025, and GSE78193 with an adjusted P value < 0.05 and |log FC| ≥ 1.0. Using the ‘ComplexHeatmap’ package, we created a differential gene clustering heatmap. The heatmap reflects the standardized expression data (z-scores) for the DEGs across all samples to provide a visual representation of expression patterns and potential clustering. In addition, we constructed volcano plots for each dataset using the ‘ggplot2’ package. The volcano plots graphically display the -log10(p-value) against the log2 fold change of all tested genes to highlight those that are significantly differentially expressed (DEGs are highlighted according to the criteria mentioned above).

### Construction of WGCNA networks and identification of modules

Weighted Gene Co-expression Network Analysis (WGCNA) serves as a computational approach for the characterization of genomic interconnections within diverse biological samples. This methodology aggregates genes based on congruent expression profiles and evaluates the relationship between gene clusters, known as modules, and particular attributes or phenotypic characteristics (20). The co-expression network was constructed using the WGCNA package in R. Genes that demonstrated statistical significance with an adjusted P value < 0.05 were selected for inclusion in the network analysis. The process began with hierarchical clustering, utilizing the “Hclust” function native to R, to detect and exclude potential outliers. Following this, the ‘pickSoftThreshold’ function was employed to determine an optimal soft-thresholding power (β), ensuring the network adhered to a scale-free topology. Using the chosen β, the ‘adjacency’ function transformed the expression similarity matrix into an adjacency matrix. Lastly, this adjacency matrix was further refined into a topological overlap matrix (TOM) using the relevant WGCNA function, thereby reducing noise and false linkages to bolster the network’s robustness. Subsequently, modules were identified using hierarchical clustering alongside the dynamic tree cut algorithm. To explore the relationship between these modules and the clinical characteristics of patients, Pearson correlation analysis was conducted, with a significance threshold set at a P value < 0.05.

### Identification of shared genes and pathway enrichment

An integrated evaluation was conducted using WGCNA and DEGs to identify core common genes. The overlap among these genes was visually represented through Venn diagrams to identify key genes for further investigation of their functional roles. Subsequently, these genes underwent thorough functional enrichment analysis. The enrichment analysis, focusing on Gene Ontology (GO) terms and pathways from the Kyoto Encyclopedia of Genes and Genomes (KEGG), utilized the “enrichplot” and “ggplot2” visualization tools in the R. To ensure the reliability of the identified relationships, a significance threshold of P < 0.05 was meticulously established.

### Feature selection using the least absolute shrinkage and selection operator

The Lasso technique, often employed in regression analysis, utilizes an ℓ1 norm regularization to promote sparsity in the solution, effectively leading to models with fewer parameters. This method not only helps in feature selection but also improves model interpretability by reducing the complexity of the model (21). A 10-fold cross-validation strategy was utilized to evaluate gene selection performance across diverse data subsets, further elucidating the generalizability of the Lasso model and the robustness of the selected genes. LASSO regression processing and drawing were conducted using Hiplot (https://hiplot.com.cn) (22). This platform facilitated the analysis and interpretation of the results obtained from the Lasso model, including the visualization of selected genes and their respective coefficients. we executed LASSO regression to ascertain the most robust predictive variables for MMD and SLE from the previously mentioned set of DEGs and the intersection of WGCNA findings. Additionally, bootstrapping was conducted with 1,000 resamples to assess the stability of the identified genes. This non-parametric technique enabled an evaluation of model accuracy through resampling from the original dataset with replacement and re-computation of the Lasso model for each bootstrap iteration.

### Expression levels of potential biomarkers and their diagnostic value

Violin plots, generated through the ggplot2 package in R, were utilized to analyze the expression patterns of key genes with a significance level established at P < 0.05. Further, the proficiency of proposed biomarkers across the datasets (GSE157628, GSE141025, GSE189993, GSE78193, and GSE154851) was assessed through the calculation of the area under the receiver operating characteristic (ROC) curve, using the pROC package in R.

### Single-sample gene set enrichment analysis

The “GSVA” R package was utilized to conduct ssGSEA for analyzing the infiltration of 28 immune cells in diseased and normal samples. Spearman’s rank correlation tests were used to examine the relationship between core genes and the levels of infiltrating immune cells, with p values calculated (P < 0.05).

## Results

### Identification of DEGs

**Figure 1:**
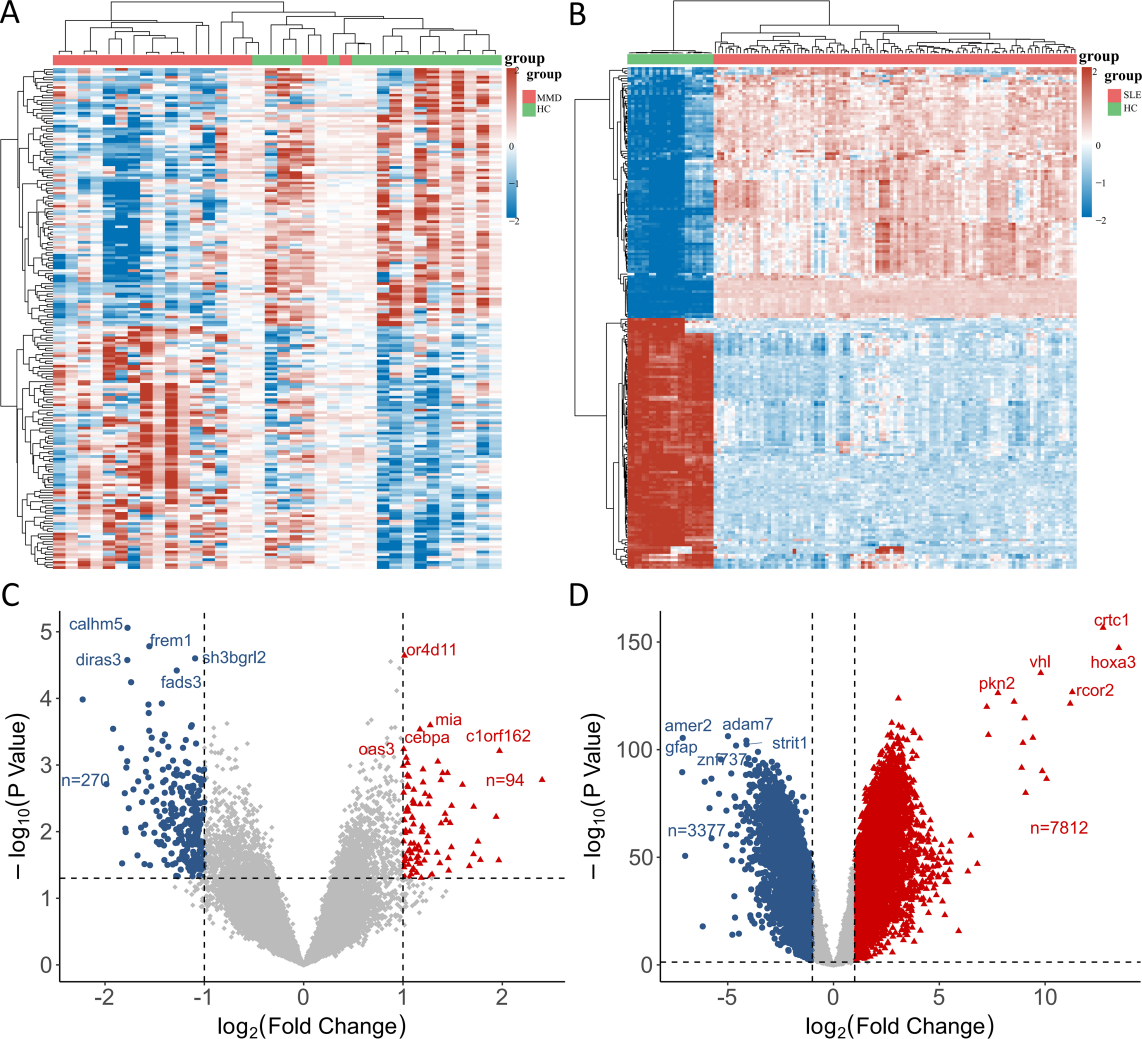
Identification of differentially expressed genes. **(A)** A heatmap of DEGs in GSE157628 and GSE141025. **(B)** A heatmap of DEGs in GSE78193. **(C)** A volcano plot of DEGs in GSE157628 and GSE141025. **(D)** A volcano plot of DEGs in GSE78193. MMD: Moyamoya disease; SLE: Systemic lupus erythematosus; HC: Healthy control.

In the GSE157628 and GSE141025 dataset for MMD, 364 DEGs were detected, comprising 94 upregulated and 270 downregulated genes. In contrast, the GSE78193 dataset for SLE revealed 11,189 DEGs, with 7,812 upregulated and 3,377 downregulated genes. Heatmaps (**Figures 1A, B**) illustrated DEGs for both diseases, while volcano plots (**Figures 1C, D**) displayed the expression patterns of the DEGs.

### Construction of WGCNA networks and identification of modules

**Figure 2:**
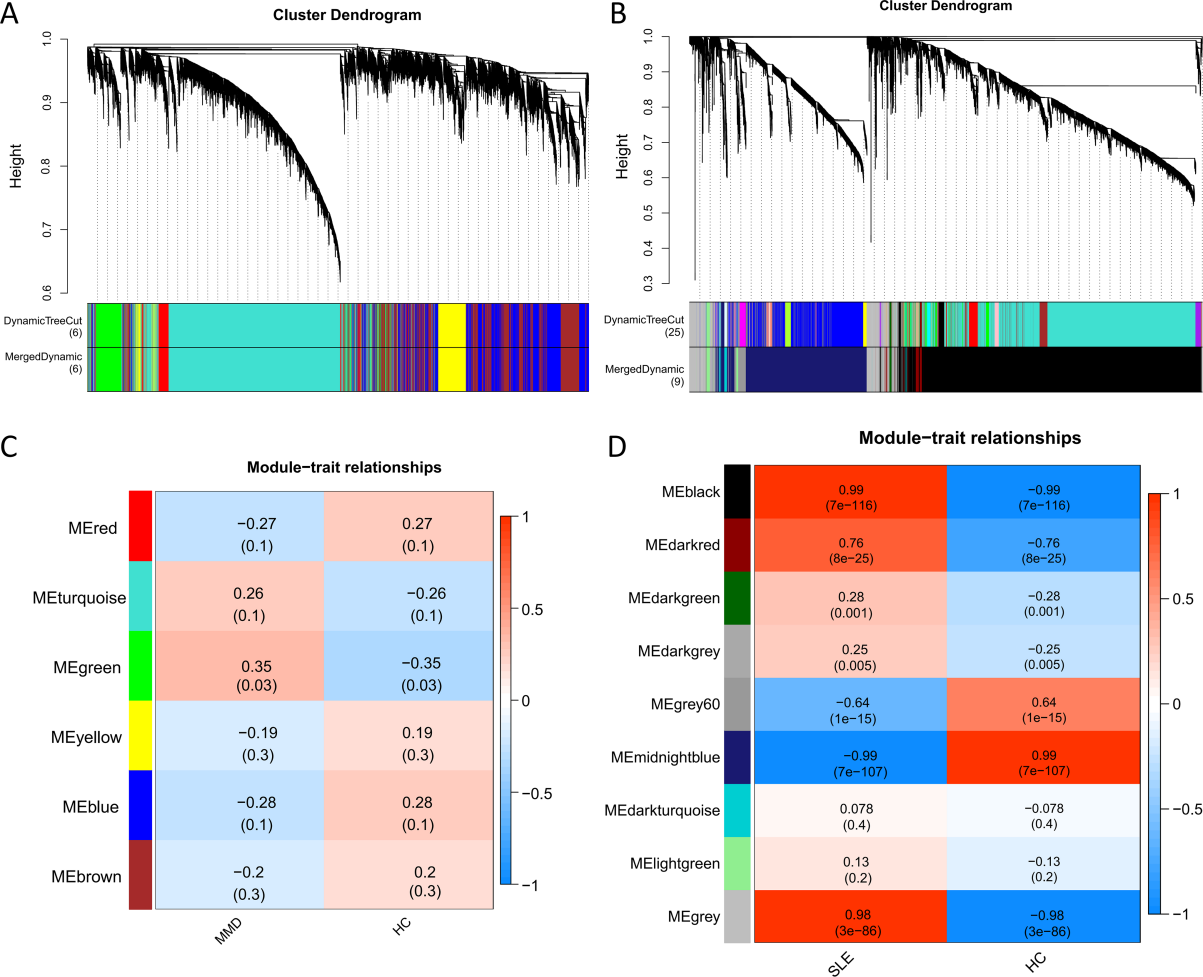
Co-expression analysis for differentially expressed genes. **(A)** Sample cluster dendrogram and trait heatmap in GSE157628 and GSE141025. **(B)** Sample cluster dendrogram and trait heatmap in GSE78193. **(C)** Heatmap of the module-trait relationships in GSE157628 and GSE141025. **(D)** Heatmap of the module-trait relationships in GSE78193. MMD: Moyamoya disease; SLE: Systemic lupus erythematosus; HC: Healthy control.

To ensure a scale-free network structure, we determined the scale-free fit index and mean connectivity. A power of β = 8 was selected for soft thresholding in GSE157628 and GSE141025, while a power of β = 26 was utilized for GSE78193. In the co-expression network analysis, 6 modules were identified in the MMD samples, and 9 modules in the SLE samples (**Figures 2A, B**).

For the investigation of genes associated with disease, we examined the relationship between modules and clinical phenotypes. In the MMD dataset (GSE157628 and GSE141025), The green module exhibited the strongest positive correlation (r = 0.35, P = 0.003), while no significant difference was observed for the strongest negative correlation. In contrast, for SLE (GSE78193), the black module demonstrated the strongest positive correlation (r = 0.99, P < 0.001), and the midnightblue module had the strongest negative correlation (r = -0.99, P < 0.001) (**Figures 2C, D**).

### Identification of shared genes, pathway enrichment, and protein-protein interaction networks

**Figure 3:**
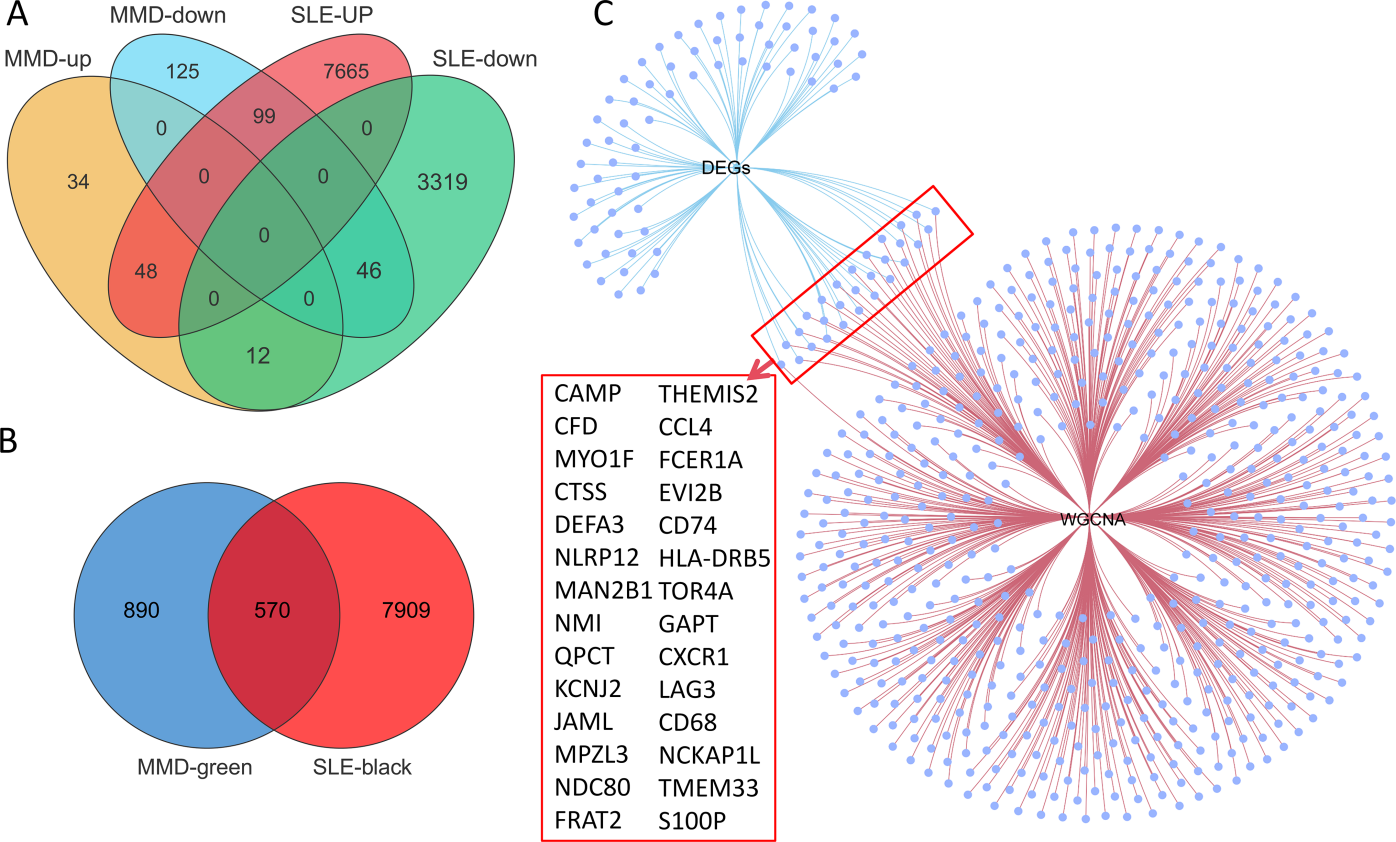
Identification of the shared genes. **(A)** Venn diagram showing an overlap of 94 DEGs between MMD and SLE. **(B)** Venn diagram shows that 570 genes overlap in the MMD and SLE modules. **(C)** Networks venn diagram showing that 28 core genes were crossed and overlapped between the genes screened by WGCNA and DEGs. MMD: Moyamoya disease; SLE: Systemic lupus erythematosus; DEG: Differentially expressed gene; WGCNA: Weighted gene co-expression network analysis.

Ninety-four overlapping DEGs were identified between MMD and SLE (**Figure 3A**). A Venn diagram was used to illustrate the overlap between the hub modules of MMD and SLE, resulting in the identification of 570 intersection genes (**Figure 3B**). Twenty-eight core genes (*CAMP, CFD, MYO1F, CTSS, DEFA3, NLRP12, MAN2B1, NMI, QPCT, KCNJ2, JAML, *MPZL3*, NDC80, FRAT2, THEMIS2, CCL4, FCER1A, EVI2B, CD74, HLA-DRB5, TOR4A, GAPT, CXCR1, LAG3, CD68, NCKAP1L, TMEM33*, and *S100P*) were identified as overlapping between the genes identified through WGCNA and DEGs. These genes may represent potential crosstalk genes between the two diseases (**Figure 3C**).

**Figure 4:**
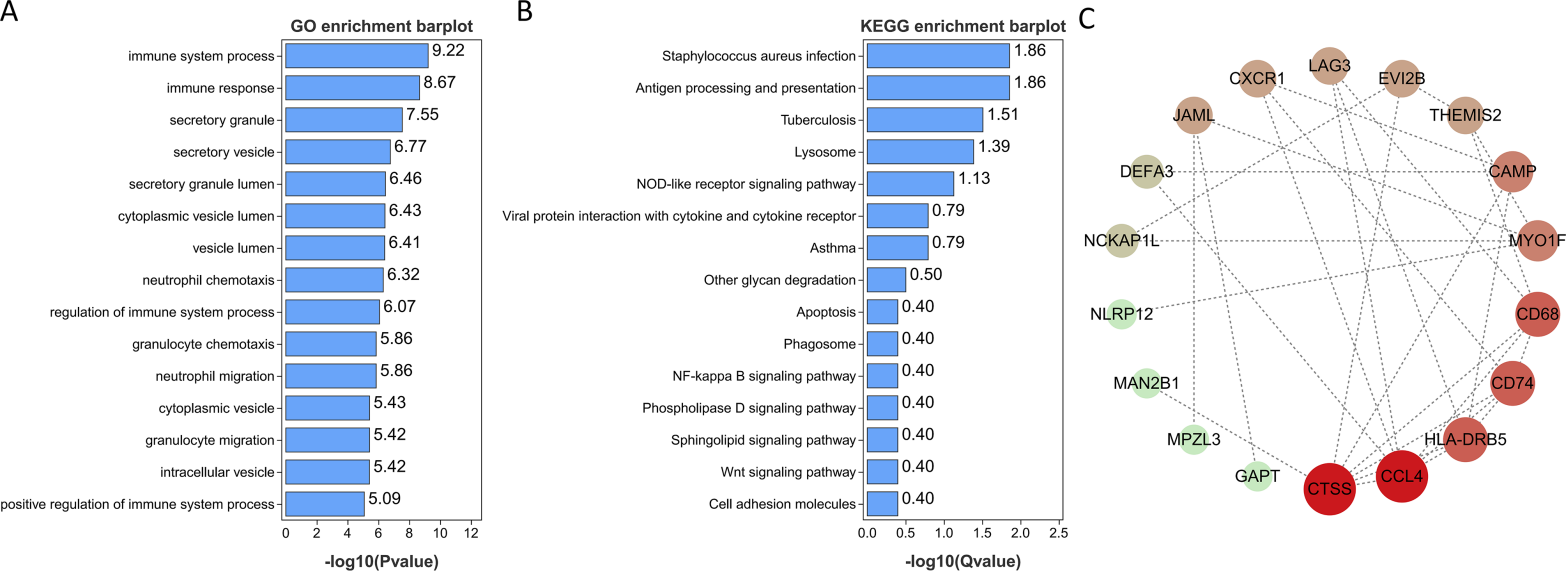
Functional enrichment analyses of the shared genes. **(A)** GO analysis of the shared genes. **(B)** KEGG pathway enrichment analysis of the shared genes. **(C)** protein-protein interaction (PPI) networks of the shared genes.

GO and KEGG enrichment analyses were conducted on the 28 genes mentioned above in order to investigate common regulatory pathways. The GO analysis revealed that these shared genes may be associated with immune system process, immune response, neutrophil chemotaxis, regulation of immune system process, and granulocyte chemotaxis (**Figure 4A**). On the other hand, the KEGG analysis indicated that these genes might primarily participate in Staphylococcus aureus infection, tuberculosis, lysosome, antigen processing and presentation, NOD-like receptor signaling pathway, viral protein interaction with cytokine and cytokine receptor, and asthma (**Figure 4B**). Finally, we employed extensive PPI network analyses using STRING databases to investigate and visualize the direct and indirect interactions of the proteins encoded by the identified genes. The more connected a protein is within the network, the more likely it is to contribute to pathway crosstalk (**Figure 4C**).

### Selection of potential shared diagnostic genes using LASSO regression

**Figure 5:**
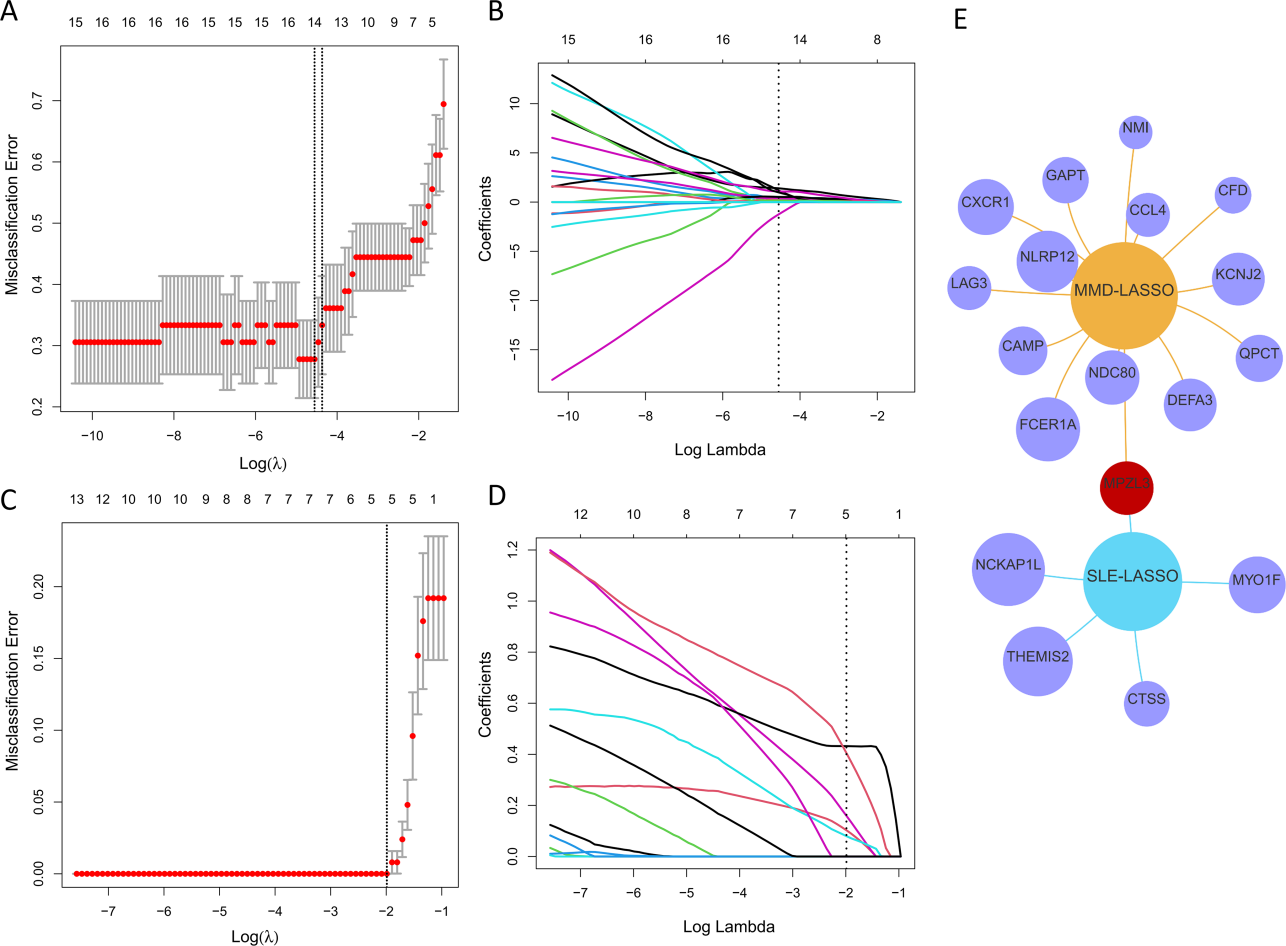
Discovering potential diagnostic genes shared across conditions using the LASSO regression. **(A)** Implementation of tenfold cross-validation for optimal tuning parameter selection, identified through log(lambda), within the GSE157628 and GSE141025 dataset. **(B)** A similar tenfold cross-validation method applied to the GSE157628 and GSE141025 dataset to determine the best log(lambda) value. **(C)** The breakdown of LASSO coefficients for the genes linked to diagnostics in the GSE78193 dataset. **(D)** LASSO coefficients for genes with diagnostic relevance within the GSE78193 dataset. **(E)** A Venn diagram illustrating shared optimal diagnostic biomarkers.

A LASSO regression was applied to pinpoint shared diagnostic biomarkers. Bootstrapping, conducted with 1,000 resamples, yielded a concordance index (c-index) of 1.000 for SLE patients and c-index of 0.868 for MMD patients. In dataset GSE157628 and GSE141025, this method revealed 14 of the 28 principal intersecting genes at an optimal lambda of 0.011 (**Figures 5A, B**). In a similar analysis of dataset GSE78193, LASSO regression pinpointed 5 out of the 28 key intersecting genes, again at optimal lambda = 0.137 (**Figures 5C, D**). Ultimately, a single gene, *MPZL3* , emerged as the most promising shared diagnostic indicator for MMD and SLE, as illustrated in **Figure 5E**. Bootstrapping, conducted with 1,000 resamples, yielded a concordance index (c-index) of 1.000 for SLE patients and c-index of 0.878 for MMD patients.

### Expression levels of candidate biomarkers and their diagnostic value

**Figure 6:**
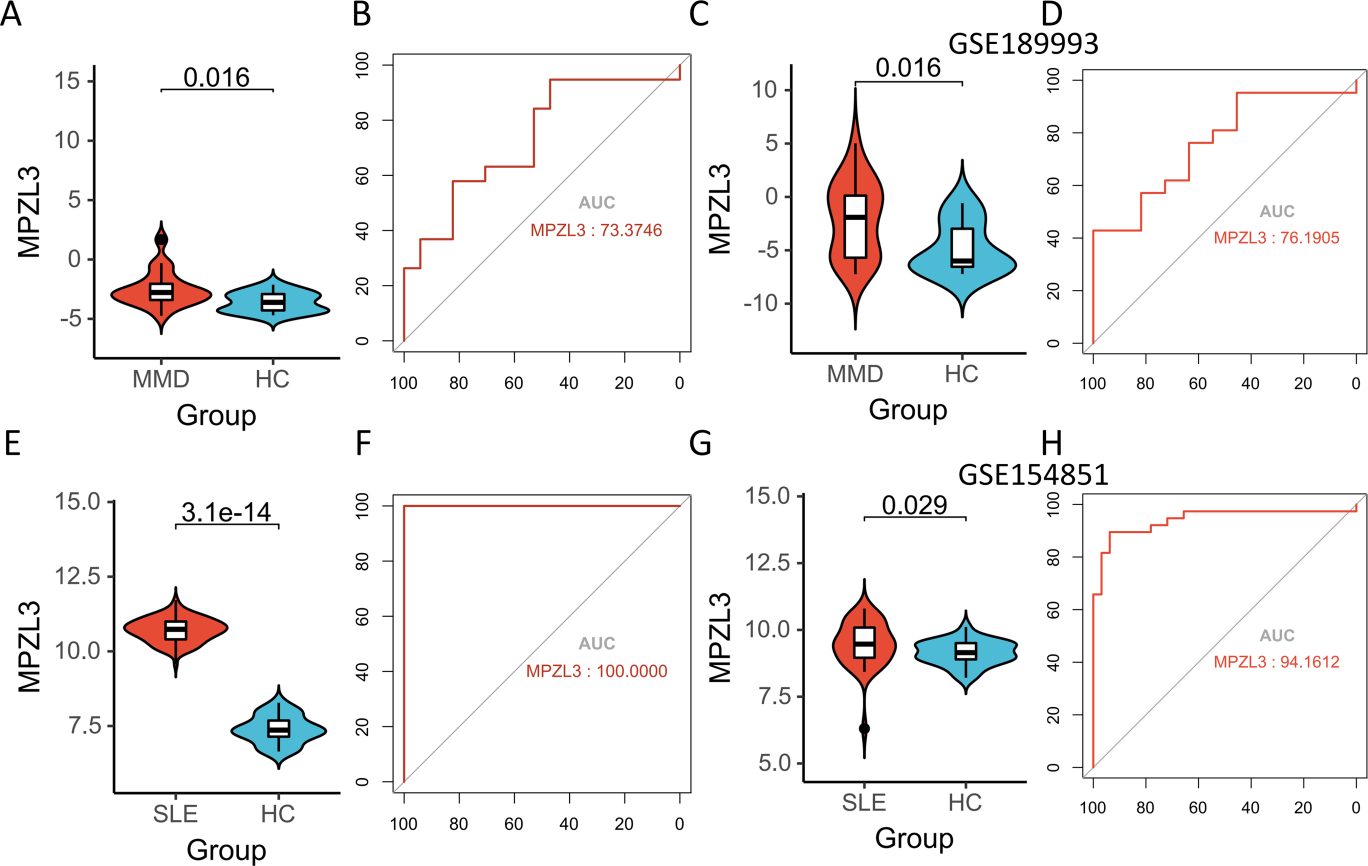
Expression pattern validation and diagnostic value. **(A)** Expression of MPZL3 in GSE157628 and GSE141025. **(B)** ROC curve of the shared diagnostic genes in GSE157628 and GSE141025. **(C)** Expression of MPZL3 in GSE189993. **(D)** ROC curve of the shared diagnostic genes in GSE189993. **(E)** Expression of MPZL3 in GSE78193. **(F)** ROC curve of the shared diagnostic genes in GSE78193. **(G)** Expression of MPZL3 in GSE154851. **(H)** ROC curve of the shared diagnostic genes in GSE154851. MMD: Moyamoya disease; SLE: Systemic lupus erythematosus; HC: Healthy control.

**Figures 6A, C, E, G** present the upregulation of *MPZL3* in both MMD and SLE, indicating their potential as biomarkers. Sensitivity and specificity assessments reveal their diagnostic potential in GSE157628 and GSE141025 datasets (**Figure 6C**), where *MPZL3* achieved an AUC of 0.734, implying substantial discriminative power. In dataset GSE78193 (**Figure 6B**), *MPZL3* exhibited considerable diagnostic capacity for SLE (AUC = 1.000) (**Figure 6F**). External validation was also conducted using datasets GSE189993 for MMD (AUC = 0.762) and GSE154851 for SLE (AUC = 0.942) (**Figures 6D, H**), where both biomarkers demonstrated strong predictive capabilities.

### Correlation between candidate biomarkers and infiltration of immune cells

**Figure 7:**
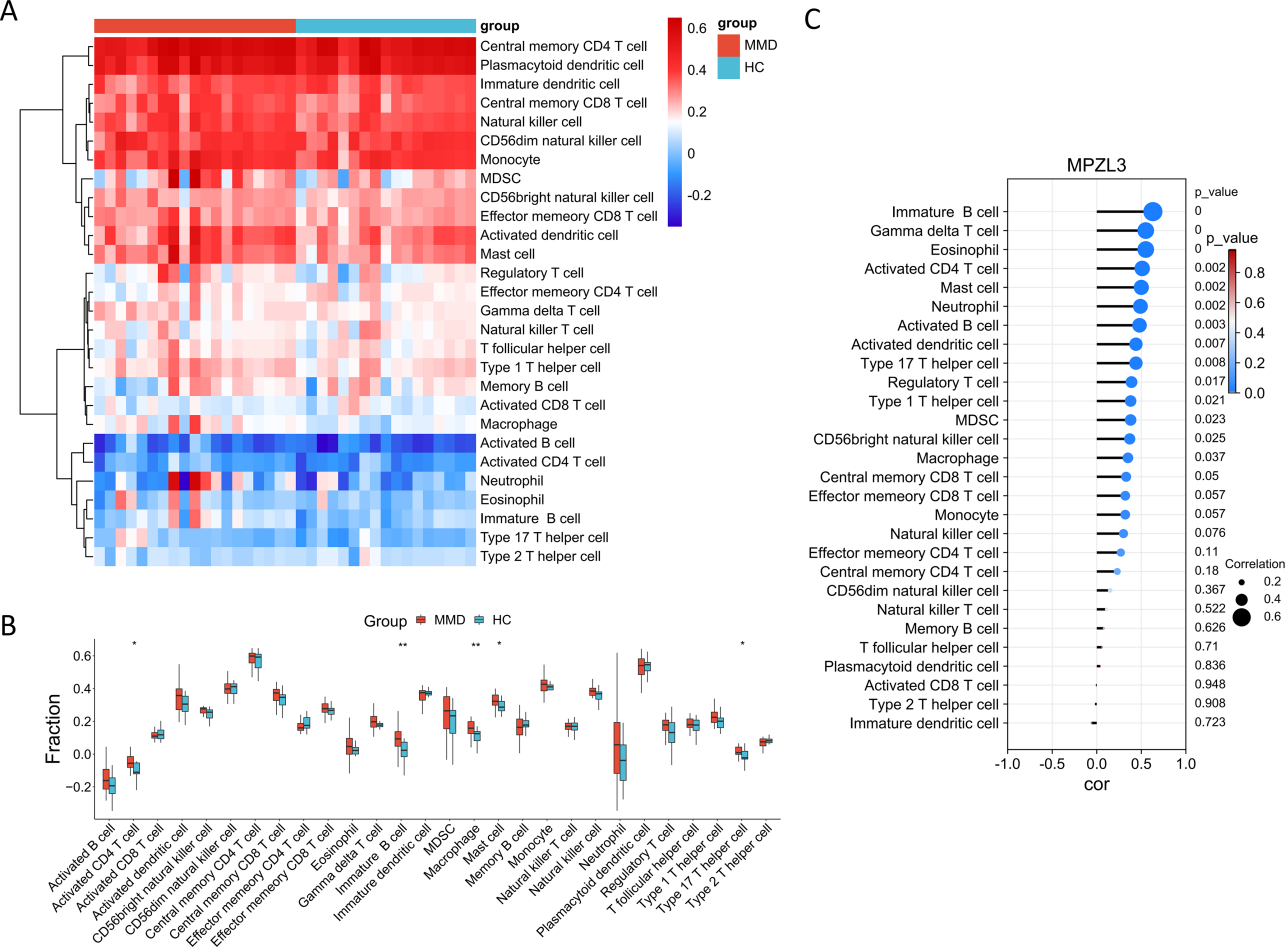
Evaluation of immune cell infiltration in relation to MMD. Heatmap **(A)** and box plot **(B)** illustrating the distribution of 28 immune cell types in the GSE157628 and GSE141025 sample. **(C)** Correlation between diagnostic genes and immune cell infiltration. MMD: Moyamoya disease; HC: Healthy control.

**Figure 8:**
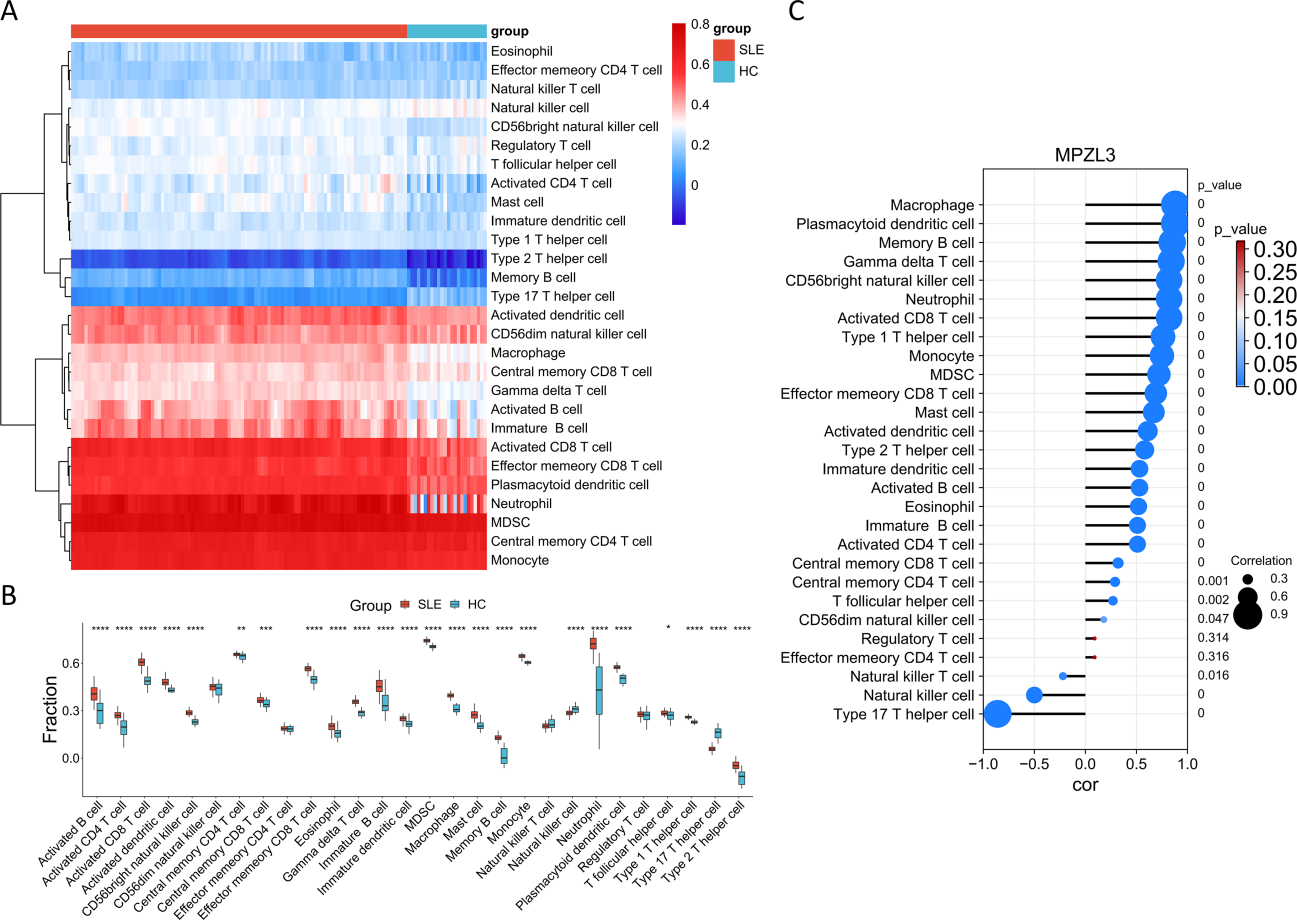
Evaluation of immune cell infiltration in relation to SLE. Heatmap **(A)** and box plot **(B)** illustrating the distribution of 28 immune cell types in the GSE78193 sample. **(C)** Correlation between diagnostic genes and immune cell infiltration. SLE: Systemic lupus erythematosus; HC: Healthy control.

We conducted a detailed analysis of immune cell infiltration in various samples. A total of 28 immune cell types were identified in the GSE157628 and GSE141025 dataset, visualized through heatmap and box plots (**Figures 7A, B**). The distribution of these 28 immune cells in the GSE78193 sample is illustrated in **Figures 8A, B**. Our findings revealed a significant increase in the infiltration of activated CD4 T cell, immature B cell, macrophages, and mast cell in both MMD and SLE. Furthermore, our correlation analysis between immune cells and candidate biomarkers indicated a positive association between activated CD4 T cell, immature B cell, macrophages, and mast cell with *MPZL3* in both MMD and SLE (**Figures 7C**, **8C**).

## Discussion

This study integrated transcriptomes of MMD and SLE, using WGCNA for the first time to investigate their shared mechanisms, uncovering potential crosstalk genes, common pathways, and associated immune cells. By intersecting DEGs with WGCNA module genes, we identified *CAMP, CFD, MYO1F, CTSS, DEFA3, NLRP12, MAN2B1, NMI, QPCT, KCNJ2, JAML, *MPZL3*, NDC80, FRAT2, THEMIS2, CCL4, FCER1A, EVI2B, CD74, HLA-DRB5, TOR4A, GAPT, CXCR1, LAG3, CD68, NCKAP1L, TMEM33*, and *S100P* as key crosstalk genes between MMD and SLE, potentially linked to immunity and inflammation regulatory activity. *MPZL3* were found to be valuable diagnostic markers. Immune infiltration analyses highlighted the significant roles of activated CD4 T cell, immature B cell, macrophages, and mast cell in the pathogenesis of both MMD and SLE.

The precise pathogenesis of MMD and SLE remains unclear; however, numerous studies have indicated that these conditions are associated with genetic factors, infections, and immune dysregulation (23, 24). This study findings provides further evidence that core crosstalk genes in MMD and SLE are associated with immune system process and immune response. The immune system process and immune response play a fundamental role in the progression and pathology of various diseases. While the exact role of the immune system in MMD remains to be fully elucidated, certain infection and autoimmune diseases have been implicated in its progression. Several studies in cohorts of MMD patients have proposed autoimmune disorders and infections as potential environmental factors that could trigger the disease. In 1980, there were already reports of 75 cases of MMD linked to leptospiral cerebral arteritis (25). In addition to infections, Graves’ disease, a prevalent autoimmune condition leading to hyperthyroidism, has been associated with MMD. Research on 170 patients with MMD, of which 25 had Graves’ disease, revealed that individuals with both conditions experienced accelerated disease advancement in MMD compared to those without Graves’ disease, resulting in a notably increased risk of stroke (26). In addition, SLE is an autoimmune disorder impacting multiple organ systems and is managed through immunomodulation and immunosuppression. Patients with SLE possess a fundamentally compromised immune system, heightened by disease activity, rendering them susceptible to infections. Immunosuppressive therapy amplifies the risk of infections, making infectious diseases a primary reason for hospitalization and mortality among SLE patients (27). Therefore, immune system process and immune response induced by infection and autoimmune diseases could potentially serve as a key factor in the shared pathophysiology of both conditions.

This study preliminarily explored the potential immune relationship between MMD and SLE, revealing significant differences in the immune patterns of the MMD and SLE groups compared to the control group. Activated CD4 T cell, immature B cell, macrophages cells showed a more significant increase in both MMD and SLE samples.

The association between T cells and MMD was initially identified in 1993, revealing that the thickened vascular intima in MMD consists primarily of smooth muscle cells and a fraction of macrophages and T cells (28). Furthermore, Leihua Weng and colleagues conducted a clinical study indicating notably elevated percentages of circulating Treg and Th17 cells in MMD patients compared to controls. Their research also highlights the significant involvement of TGF-β in the advancement of MMD (29). These findings align with the results from our ssGSEA analysis. Immunohistochemical analysis revealed B cells were infrequently observed in the patients (28). Remarkably, we observed a notable rise in immature B cell in MMD. In addition, collaboration between activated naïve B cells and CD4+ T cells facilitated SLE development by enhancing the differentiation of pathogenic T cells (Th2 and Th17) as well as the production of autoantibodies (30). Recent clinical studies have shown a positive association between the quantity of monocytes expressing M1 macrophage-like markers (CD163-CD14+) in the peripheral blood of children with lupus and the severity of childhood-onset SLE (31). Similarly, agents promoting M1 macrophage polarization may worsen inflammatory disorders like lupus (32). Thus, it is speculated that SLE could aggravate the onset and advancement of MMD through the activation of T and monocytes cells.

To mitigate the risk of overfitting and to enhance the reliability of performance metrics, it is crucial to incorporate a substantial number of samples in clinical biomarker identification studies (33). In our research, we included 19 MMD samples from the GSE157628 and GSE141025 dataset and 101 SLE samples from the GSE78193 dataset. The effectiveness of a biomarker can be measured using the area which ranges from 0 to 1 (34). A higher AUC value signifies a more accurate diagnostic test. In our analysis, ROC assessment revealed that the biomarker *MPZL3* had an AUC of 0.734 for MMD prediction and 1.000 for SLE prediction. Consequently, our findings suggest that both *MPZL3* demonstrate strong predictive capabilities for MMD and SLE.

As a significant gene facilitating the interaction between MMD and SLE, *MPZL3* is a nuclear-encoded protein that is predominantly localized in the mitochondria. It possesses an immunoglobulin-like V-type structure and plays a crucial role in regulating epithelial cell differentiation, lipid metabolism, reactive oxygen species (ROS) generation, glycemic control, and energy expenditure (35). *MPZL3* contains an immunoglobulin domain and functions as a cell adhesion molecule, regulating the recruitment of immune cells during inflammation. *MPZL3* may play a role in the inflammatory response to dietary fat intake (36). A positive correlation was identified between the infiltration of CD8+ T cells, CD4+ T immune cells, B cells, and other immune cells, and the expression of *MPZL3* in breast invasive carcinoma (BRCA) (37). Elevated levels of *MPZL3* expression have been associated with the activation of immune cell signaling pathways. Previous research has suggested that *MPZL3* expression in immune cells such as dendritic cells, CD4, and CD8 central memory and effector T cells supports its potential role in immunity. Moreover, mutations in the conserved V-type domain of *MPZL3* can impact immune function, potentially leading to immunodeficiencies (38). *MPZL3* and *FDXR* collaborate to elevate ROS levels, promoting epidermal differentiation. The differentiation induced by ROS is contingent upon *MPZL3* enhancing *FDXR* enzymatic activity. The generation of ROS by the mitochondrial proteins *MPZL3* and *FDXR* is crucial for driving epidermal differentiation (39). In summary, there is a potential involvement of inflammation and immunity mediated by *MPZL3* in the interplay between MMD and SLE; however, additional data is required to confirm this association. This will emphasize the shared immune-level pathophysiology and could be vital in comprehending the link between MMD and SLE.

Our study exhibits multiple strengths. Initially, we employed a comprehensive and intricate bioinformatics analysis as a novel approach to explore the association between the two diseases. The utilization of the LASSO regression algorithm facilitated the identification of potential shared diagnostic genes. Validation using external datasets enhanced the accuracy of our predictions. However, there are some limitations in our research. Our findings were based on distinct patient cohorts and lacked validation within the same individuals. Establishing a model that combines MMD and SLE is essential to confirm the potential relationship between these conditions in future studies. Additionally, this study did not consider data on age, gender, medication, and patient comorbidities, which could impact the reliability of the current results. Finally, our study was not experimentally validated due to the absence of dependable cell and animal models.

### Conclusion

This study is pioneering in its use of bioinformatics tools to explore the close genetic relationship between MMD and SLE. The genes *CAMP, CFD, MYO1F, CTSS, DEFA3, NLRP12, MAN2B1, NMI, QPCT, KCNJ2, JAML, MPZL3, NDC80, FRAT2, THEMIS2, CCL4, FCER1A, EVI2B, CD74, HLA-DRB5, TOR4A, GAPT, CXCR1, LAG3, CD68, NCKAP1L, TMEM33*, and *S100P* have been identified as key crosstalk genes that connect MMD and SLE. Activation of T and monocytes cells-mediated immune responses are proposed to play a significant role in the association between MMD and SLE.

## Data Availability

The datasets presented in this study can be found in online repositories. The names of the repository/repositories and accession number(s) can be found in the article/**Supplementary Material**.
