# Supplementary figures and images for: Exploring the transcriptomic landscape of moyamoya disease and systemic lupus erythematosus: insights into crosstalk genes and immune relationships
Source: Front Immunol. 2024 Sep 3;15:1456392. doi: 10.3389/fimmu.2024.1456392 (PMC11405312; doi:10.3389/fimmu.2024.1456392)

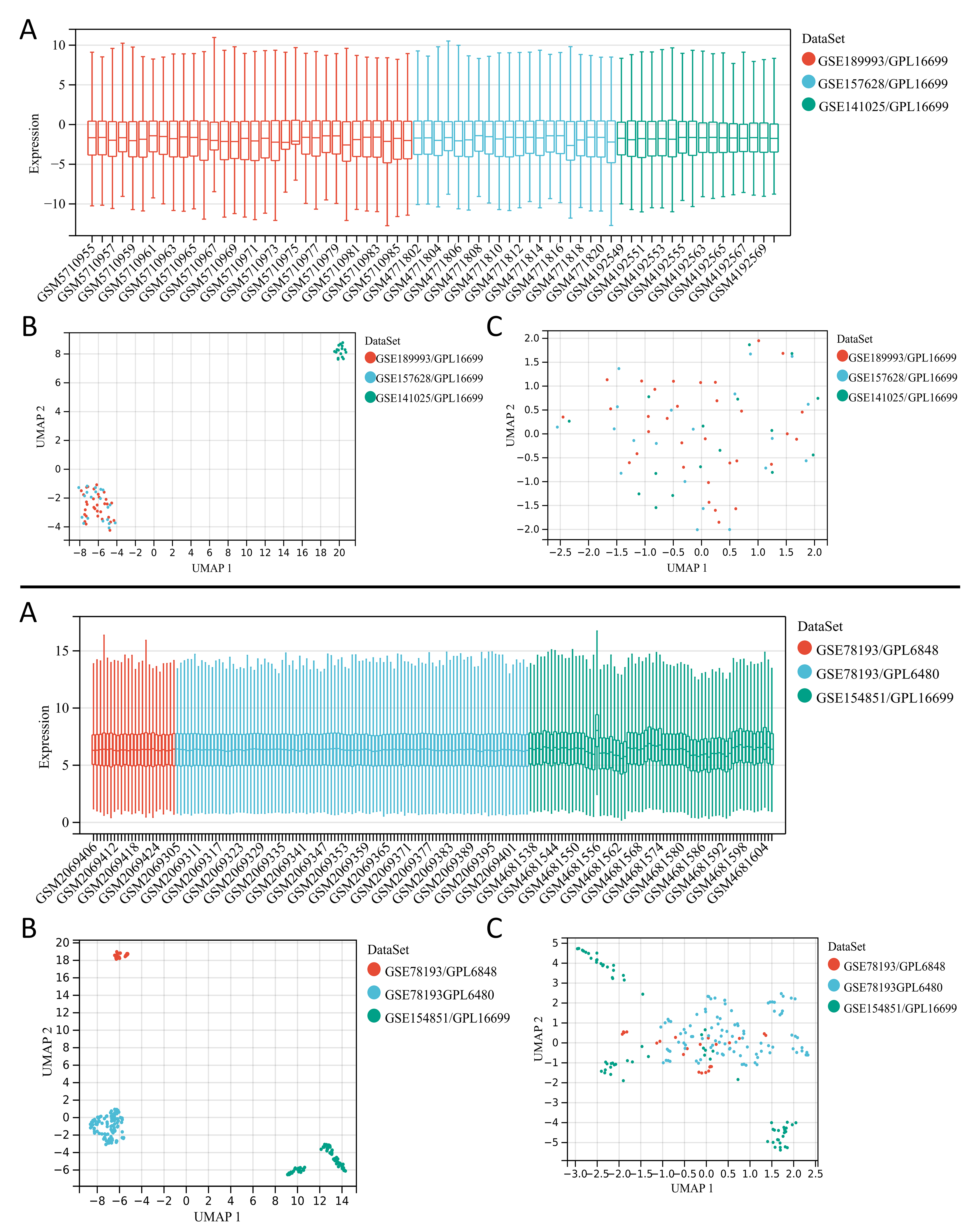

Supplement: Supplementary file 1 [file Image1.tif]
